# Developmental differences in the expression of ABC transporters at rat brain barrier interfaces following chronic exposure to diallyl sulfide

**DOI:** 10.1038/s41598-019-42402-8

**Published:** 2019-04-12

**Authors:** Liam M. Koehn, Katarzyna M. Dziegielewska, Kjeld Møllgård, Elodie Saudrais, Nathalie Strazielle, Jean-Francois Ghersi-Egea, Norman R. Saunders, Mark D. Habgood

**Affiliations:** 1 Department of Pharmacology and Therapeutics, The University of Melbourne, Melbourne, Victoria, Australia; 2 Institute of Cellular and Molecular Medicine, University of Copenhagen, Copenhagen, Denmark; 3 IBIP facility and Fluid team, Lyon Neuroscience Research center, NSERM U1028 CNRS UMR5292, Université de Lyon-1, Lyon, France; 4 Brain-I, Lyon, France

## Abstract

Many pregnant women and prematurely born infants require medication for clinical conditions including cancer, cardiac defects and psychiatric disorders. In adults drug transfer from blood into brain is mostly restricted by efflux mechanisms (ATP-binding cassette, ABC transporters). These mechanisms have been little studied during brain development. Here expression of eight ABC transporters (*abcb1a*, *abcb1b*, *abcg2*, *abcc1*, *abcc2*, *abcc3*, *abcc4*, *abcc5*) and activity of conjugating enzyme glutathione-s-transferase (GST) were measured in livers, brain cortices (blood-brain-barrier) and choroid plexuses (blood-cerebrospinal fluid, CSF, barrier) during postnatal rat development. Controls were compared to animals chronically injected (4 days, 200 mg/kg/day) with known *abcb1a* inducer diallyl sulfide (DAS). Results reveal both tissue- and age-dependent regulation. In liver *abcb1a* and *abcc3* were up-regulated at all ages. In cortex *abcb1a/b*, *abcg2* and *abcc4/abcc5* were up-regulated in adults only, while in choroid plexus *abcb1a* and *abcc2* were up-regulated only at P14. DAS treatment increased GST activity in livers, but not in cortex or choroid plexuses. Immunocytochemistry of ABC transporters at the CSF-brain interface showed that PGP and BCRP predominated in neuroepithelium while MRP2/4/5 were prominent in adult ependyma. These results indicate an age-related capacity of brain barriers to dynamically regulate their defence mechanisms when chronically challenged by xenobiotic compounds.

## Introduction

Most clinicians adopt a cautious approach when prescribing medications to pregnant women due to concerns that these compounds may reach the fetus, where they could adversely affect specific organs such as the brain^1^. There are many clinical situations, however, in which it is not reasonable to withhold drug therapy from a pregnant woman (e.g. epilepsy, cancer or severe psychiatric disorders). There are no clinical trial data available to doctors on which to base treatment advice, as such trials have not been carried out on pregnant women or neonates for obvious ethical reasons^2^. In the absence of human data animal experiments provide a means of understanding the biology and functional capacity of the protective mechanisms present in the developing brain. Understanding which classes of drugs, under what conditions and at which stages of development are likely to be restricted or excluded from entering the developing brain would allow for safer prescription of drugs deemed to be clinically essential for pregnant women and newborns, especially when premature^3^.

Molecules, including drugs, are restricted from crossing blood-brain interfaces by a number of cellular and metabolic defence mechanisms^4–6^. Lipid-insoluble compounds (unable to diffuse through cellular membranes) are prevented from intercellular transfer by tight junctions that link barrier-forming cells^5,7^. Lipid soluble compounds, that in principle are able to traverse cellular membranes, can be restricted from crossing the barrier by a range of mechanisms that include enzymatic conjugation and efflux transportation. At the blood-brain barrier interface in adults the main efflux transporters that have been identified are P-glycoprotein (PGP, *abcb1*, MDR1), breast cancer resistance protein (BCRP, *abcg2*) and multidrug resistance-associated proteins 2 and 4 (MRP2, *abcc2*; MRP4, *abcc4*)^4,8^. At the choroid plexus blood-CSF (cerebrospinal fluid) interface, multidrug resistance-associated protein 1 (MRP1, *abcc1*) is the predominant efflux transporter, with MRP4 (*abcc4*) and BCRP (*abcg2*) also identified previously^8,9^. ABC-transporters (PGP; *abcb1a/b* and BCRP; *abcg2*) have also been identified at the arachnoid barrier between the blood and outer CSF^10^.

In clinical practice medications are often administered chronically in order to treat a disease. It has been suggested that the efflux capacity of blood-brain barriers can dynamically respond to prolonged drug exposure by increasing the amount/activity of ABC transporters present, thereby limiting subsequent drug transfer into the brain. For the blood-brain interfaces, where efflux transporter expression has been previously described, determining whether or not these levels can alter over the course of a therapy will be integral to our understanding of the total drug transfer over time.

Repeat exposure to dexamethasone or pregnenolone-16α-carbonitrile (PCN) in the adult rat^11^ has been shown to increase PGP (*abcb1a/b)* expression at the blood-brain barrier. A large-scale investigation in mice of 15 compounds has highlighted diallyl sulfide (DAS) as the most prominent inducer of *abcb1a/b* (PGP) expression at this barrier^12^. Both *ex vivo* and *in vivo* adult mouse models have also revealed an increase in *abcg2* (BCRP) expression in the mouse blood brain barrier in response to clofibrate administration^13^. These studies, however, have only been completed in adults but not in younger animals. It is possible that not all stages of development have the same capacity to dynamically regulate efflux transporters, meaning that repeat drug exposure may pose a greater risk at certain developmental stages.

Efflux transporters bind substrates either directly (PGP) or as conjugates (MRPs and BCRP) following phase II conjugation to attach an appropriate binding motif^14^. The series of conjugating enzymes therefore play a significant role in the mechanism of efflux as either the enzyme or the transporter could be the limiting factor in the removal of certain xenobiotics that require conjugation before efflux. The presence and activity of certain conjugating enzymes, such as glutathione-s-transferase (GST), have been previously described in cortical endothelial cells and choroid plexus epithelial cells in both the rat and human^15,16^. Glutathione conjugation at the blood-CSF barrier is known to be important in xenobiotic exclusion from the brain, especially during development^17^.

Inducers known to up-regulate *abcb1a* (PGP) and *abcc2* (MRP2) expression have been shown to also up-regulate GST at the adult rat blood-brain barrier^18^. Whether or not this ability to up-regulate the expression of efflux associated conjugation genes in response to drug exposure also occurs at earlier stages of development is yet to be investigated. A complex examination of both efflux transporter and conjugating enzyme regulation would provide a comprehensive understanding of barrier response to chronic drug exposure.

ABC-transporter studies have previously been largely focused on their presence or absence at the two main blood-brain interfaces: the blood-brain barrier proper (formed by tight junctions between endothelial cells in brain vasculature) and the blood-CSF barrier (formed by tight junctions between epithelial cells of the choroid plexus). Both have been extensively reviewed^4–6,19–21^. However, additional barrier interfaces are known to be present at other sites within the brain^4–6^, including a CSF-brain barrier that is characteristically present only during early stages of development. This interface controls the exchange of molecules between the CSF and brain tissue at the level of the neuroepithelial ventricular lining^22^. It has been proposed that during early brain development the main route of access into the brain is via the CSF^23,24^. Thus establishing which efflux transporters are present at this interface is important for determining the overall blood to brain transfer of drugs.

The present study examines the regulation of efflux capacity at brain cortices (as a site of the blood-brain barrier) and choroid plexuses (site of the blood-CSF barrier) early in development and identifies the efflux transporters that are present at the developmentally specific CSF-brain interface. The results show not only that one inducer (diallyl sulfide, DAS) can up-regulate a range of efflux transporters, but also that the profile of up-regulation is both tissue specific and age-dependent. DAS induced up-regulation of brain cortical efflux transporters *abcb1a/b* (PGP), *abcg2* (BCRP) and *abcc4/5* (MRP4/5), but only in the adult. At the blood-CSF barrier *abcb1a* (PGP) and *abcc2* (MRP2) up-regulation was observed at postnatal day 14 (P14), but not at adult or P4. These instances of efflux transporter up-regulation occur independently of up-regulation of conjugating enzymes such as GST. In addition, liver showed up-regulation of *abcb1a* (PGP) and *abcc3* (MRP3) at all ages indicating that age related differences in induction are tissue specific. Studies of the presence and developmental changes in efflux transporters at the ventricular interface between the CSF and brain parenchyma have not been comprehensively described previously. This interface is important in regulating the exchange of molecules between ventricular CSF and brain tissue, especially during early brain development. Together the results from this study describe the expression and regulation of the protective mechanisms present at three main brain interfaces over the course of postnatal rat brain development.

## Results

Throughout this paper the ABC transporter terminology will reflect the experiment described. For RT-qPCR, genes will be listed with common protein names in brackets, e.g. *abcc1* (MRP1), whereas for immunohistochemistry the protein will be listed with associated gene in brackets, e.g. MRP1 (*abcc1*). In addition, we refer to the treatment regime as chronic when the animals are injected over several consecutive days.

### Expression of Efflux Transporters (RT-qPCR)

**Figure 1:**
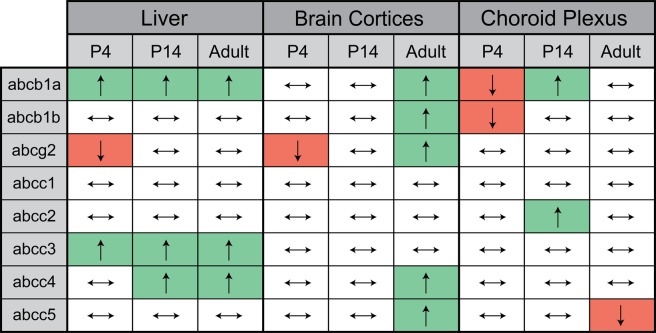
A summary of results from the RT-qPCR analysis of ABC transporter regulation following chronic diallyl sulfide (DAS; 200 mg/kg) treatment. The relative expression of every gene (for each tissue and age) was determined as 2^−ΔCt^ with respect to an average of two housekeeping genes (*β-actin* and *ppib*). Transporter levels were compared between treated and control animals and results illustrated as green (↑) indicating significant up-regulation following treatment (p < 0.05), white (↔) indicating no significant change between groups and red (↓) indicating significant down-regulation (p < 0.05). Significance was determined by two-tailed students t-test for normal distributions and Mann-Whitney U for non-normal data. Note that expression levels were affected differently by DAS treatment in different tissues and also for some tissues at different ages.

The expression of eight main ABC efflux transporters was measured in liver, brain cortices (as a reflection of blood-brain barrier) and lateral ventricular choroid plexus (site of blood-CSF barrier) of chronically DAS-treated animals and untreated age-matched controls. Three postnatal age groups were investigated: P4, P14 and adult. The expression levels in the liver were used as an internal control in individual animals to confirm that the injection protocol of DAS was appropriate and sufficient to up-regulate liver expression. DAS has been previously shown to up-regulate *abcc3* (MRP3) in rat livers^25,26^. A summary of all RT-qPCR results is shown in Fig. 1. All RT-qPCR results can be found in Supplementary Fig. S1 for liver, supplementary Fig. S2 for brain cortices and Supplementary Fig. S3 for choroid plexuses.

#### Liver

**Figure 2:**
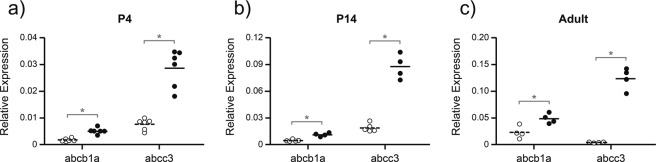
Hepatic expression of *abcb1a* (PGP) and *abcc3* (MRP3) for both untreated control animals (open symbols) and chronically DAS treated (200 mg/kg; filled symbols) animals. Relative expression was calculated as 2^−ΔCt^ with respect to an average of two housekeeping genes: *β-actin* and *ppib*. Ages investigated were P4 (**a**; n = 6), P14 (**b**; n = 4–5) and adult (**c**; n = 4), referring to the age when tissue was collected following the completion of the treatment protocol. * indicates a significant difference (p < 0.05) between treatment groups for each age and transporter. Note different scales on the y-axis of (**a**–**c)**.

The hepatic expression levels of *abcb1a* (PGP) and *abcc3* (MRP3) for both the DAS-treated and control groups at each postnatal age are shown in Fig. 2. There was a significant increase in *abcb1a* (PGP) expression in the DAS-treated group compared to controls in the adult (p = 0.012), P14 (p = 0.0001) and P4 (p = 0.00003) groups. There was also a significant increase in *abcc3* (MRP3) expression in the DAS-treated group compared to controls in the adult (p = 0.001), P14 (p = 0.00002) and P4 (p = 0.0004) groups. Thus DAS treatment included a similar pattern of hepatic ABC transporter (*abcc3* and *abcb1a*) up-regulation at all three postnatal ages investigated. Additional increases of *abcc4* (MRP4) were observed in the adult (p = 0.028) and P14 (p = 0.011) liver along with a decrease in *abcg2* (BCRP) expression at P4 (p = 0.006), although the level of expression of these transporters was comparatively low. The expression of *abcb1b*, *abcc1*, *abcc2*, or *abcc5* in livers was not affected by DAS exposure (Fig. 1, Supplementary Fig. S1). Thus liver expression acted as an internal positive control to confirm that all animals used in the study had received a dose of DAS that was sufficient to induce ABC transporter up-regulation.

#### Brain cortices (as a reflection of blood-brain barrier)

**Figure 3:**
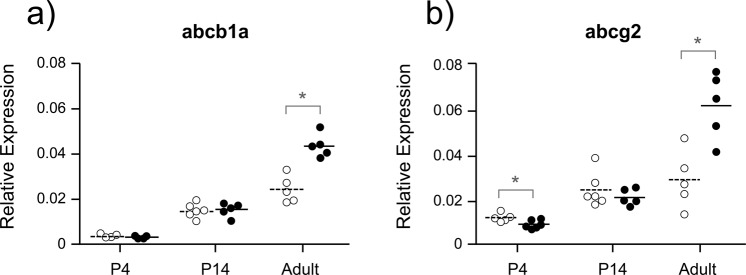
Expression of *abcb1a* (**a**; PGP) and *abcg2* (**b**; BCRP) in cortical samples of untreated control (open symbols) and chronically DAS treated (200 mg/kg; filled symbols) animals. Relative expression was calculated as 2^−ΔCt^ with respect to an average of two housekeeping genes: *β-actin* and *ppib*. Ages investigated were P4 (n = 5–6), P14 (n = 5) and adult (n = 5), referring to the age when tissue was collected following the completion of the treatment protocol. * indicates a significant difference (p < 0.05) between treatment groups for each age and transporter.

The expression levels of *abcb1a* (PGP) and *abcg2* (BCRP) in the cortices of both the DAS treated and control groups for each age are shown in Fig. 3. In the adult group there was a significant increase in *abcb1a/b* (PGP) expression in the DAS treated group compared to controls (p = 0.0298; p = 0.0037). In contrast, there was no significant increase in *abcb1a/b* (PGP) expression in the P4 or P14 age groups following DAS treatment (Fig. 3). Significant increases in expression of *abcg2* (BCRP; p = 0.0056), *abcc4* (MRP4; p = 0.023) and *abcc5* (MRP5; p = 0.017) were also only observed in the adult brain compared to controls (Fig. 1). DAS treatment, therefore, was capable of increasing cortical *abcb1a/b* (PGP), *abcg2* (BCRP) and *abcc4-5* (MRP) expression in adults, but not in animals at earlier stages of development. No significant up-regulation was observed for *abcc1*, *abcc2 or abcc3* at any of the ages investigated (Fig. 1, Supplementary Fig. S2). There was, however, a significant decrease in *abcg2* (BCRP) expression in the P4 group (p = 0.029; Fig. 3). The appropriateness of using the whole cortex for assessing blood-brain barrier efflux mechanisms is considered in the Discussion.

#### Lateral ventricular choroid plexuses (blood-CSF barrier)

**Figure 4:**
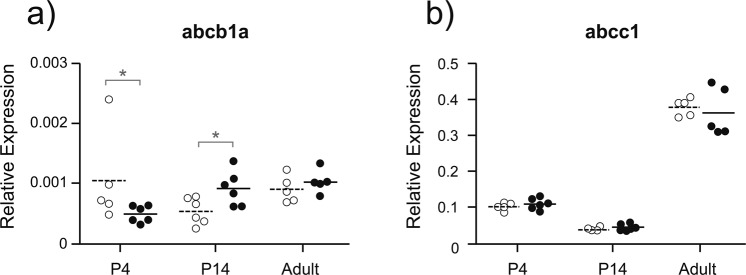
Expression of *abcb1a* (**a**; PGP) and *abcc1* (**b**; MRP1) in lateral ventricular choroid plexus (LVCP) of untreated control (open symbols) and chronically DAS treated (200 mg/kg; filled symbols) animals. Relative expression was calculated as 2^−ΔCt^, with respect to an average of two housekeeping genes: *β-actin* and *ppib*. Ages investigated were P4 (n = 6), P14 (n = 6) and adult (n = 5) referring to the age when tissue was collected following the completion of the treatment protocol. * indicates a significant difference (p < 0.05) between treatment groups for each age and transporter. Note different scales on the y-axis of (**a**,**b**).

The expression of *abcb1a* (PGP) and *abcc1* (MRP1) in the lateral ventricular choroid plexus is shown in Fig. 4. Despite DAS-treated adult animals exhibiting significantly up-regulated expression of *abcb1a* (PGP) in the cortex (Fig. 3), no up-regulation of *abcb1a* was observed in the lateral ventricular choroid plexus. Notably, the level of *abcb1a* (PGP) expression in the choroid plexus (Fig. 4) was considerably lower than in cerebral cortex (Fig. 3). At younger ages, however, a significant up-regulation of *abcb1a* (PGP) occurred at P14 (p = 0.030) whereas a significant down-regulation was observed at P4 (p = 0.036). Expression of *abcb1b* (PGP) was also down-regulated at P4 (p = 0.004) with no change observed at P14 or adult (Fig. 1). Expression of *abcc5* (MRP5) was down-regulated by DAS in adults (p = 0.012), but no change in expression levels at P4 or P14. Expression of *abcc2* (MRP2) was up-regulated at P14 (p = 0.028), but not at any other age. No up- or down-regulation was observed for the primary choroid plexus efflux transporter *abcc1* (MRP1; Fig. 4) or for *abcg2* (BCRP), *abcc3* (MRP3) *and abcc4* (MRP4; Fig. 1, Supplementary Fig. S3). Results for the lateral ventricular choroid plexus indicate a larger age-related variation in the response to DAS than was apparent for the cortex.

### Glutathione-S-transferase (GST) Activity

**Figure 5:**
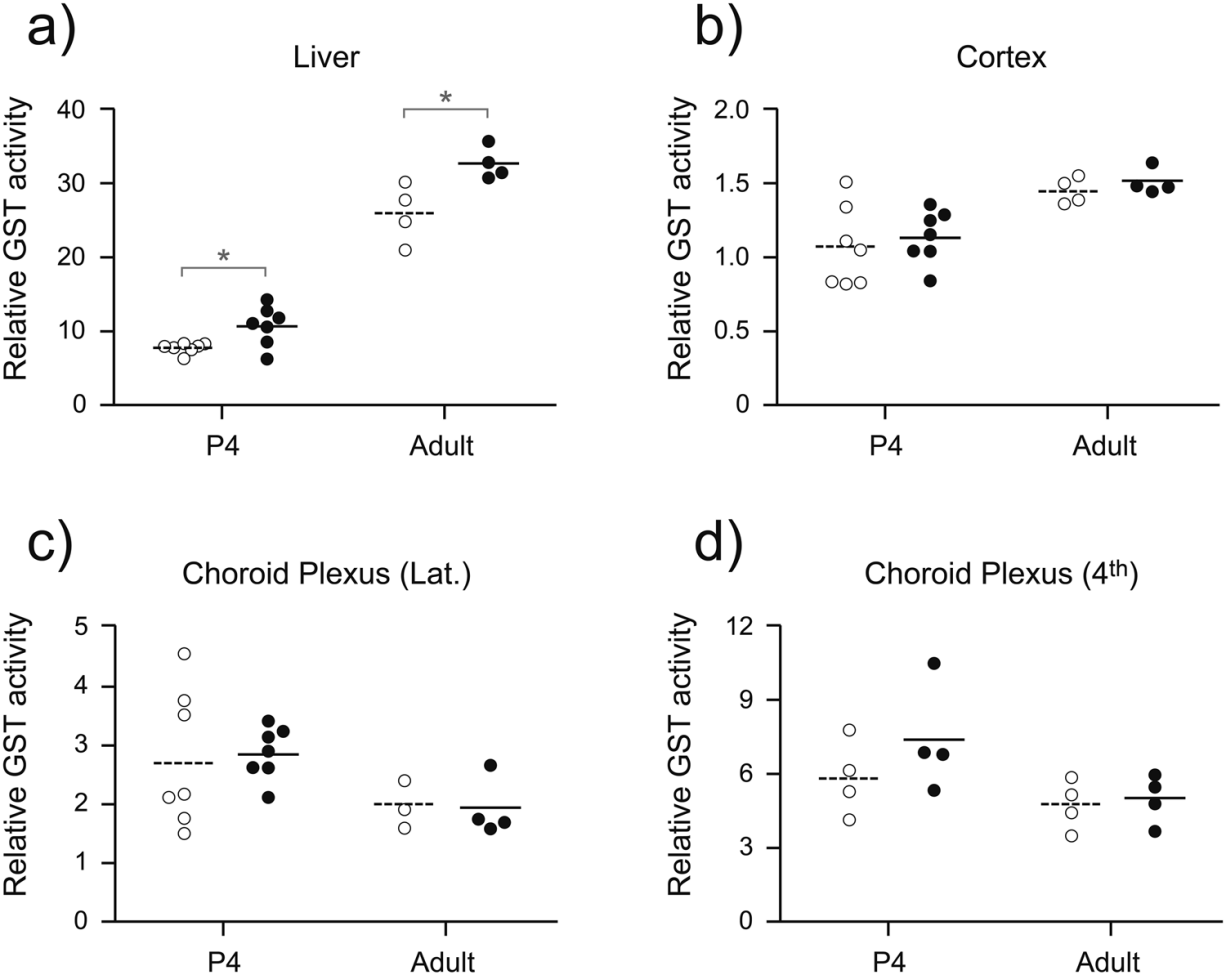
Glutathione-S-transferase (GST) activity in the liver (**a**), cortex (**b**), lateral choroid plexus (**c**) and 4^th^ ventricular choroid plexus (**d**) of both untreated control (open symbols) or chronically DAS treated (200 mg/kg; closed symbols) animals. Relative GST activity was calculated as nmol/min/mg. Ages investigated were P4 (n = 4–7) and adult (n = 4), referring to the age when tissue was collected following the completion of the treatment protocol. Tissue for choroid plexus measurements were pooled from 3 animals. * indicates a significant difference (p < 0.05) between treatment groups for each age and tissue. Note different scales on the y-axis of (**a**–**d**).

The activity of GST was measured to determine if DAS was able to induce the expression of one of the conjugating enzymes that contribute, along with efflux transporters, to xenobiotic efflux. Hepatic GST activity was increased at both postnatal ages investigated in response to DAS treatment, P4 (p = 0.012) and adult (p = 0.024; Fig. 5). DAS treatment did not result in significant increase in GST activity compared to controls in the cortex, lateral ventricular choroid plexus or 4^th^ ventricular choroid plexus at any age investigated (Fig. 5). DAS treatment, therefore, appears to have a tissue selective effect, increasing GST expression in the liver, but not affecting expression levels at the choroid plexuses and brain parenchyma.

### Immunohistochemical analysis of ABC transporters at the CSF-brain interface

As outlined in the Methods, the interface between the CSF and the brain was included in the cerebral cortex samples for RT-qPCR and therefore would have contributed to the overall sample of RNA extracted. This cellular layer is known to change during development^22^. Morphologically it changes from a neuroepithelium (neuroependyma) in early stages of development to the ependymal layer in adults. It also changes its permeability properties. There is a diffusional restraint comprised of “strap” junctions during neuroepithelial stages whereas intercellular diffusion is unrestricted in the ependymal layer due to lack of strap junctions and presence of gap junctions^22^. Glut9^27^ and MRP1^28^ have both been identified at the CSF-brain barrier previously. A comprehensive study has not been completed to investigate which ABC transporters are present at this interface and whether they change during development. This information is important in the interpretation of RT-qPCR results.

**Figure 6:**
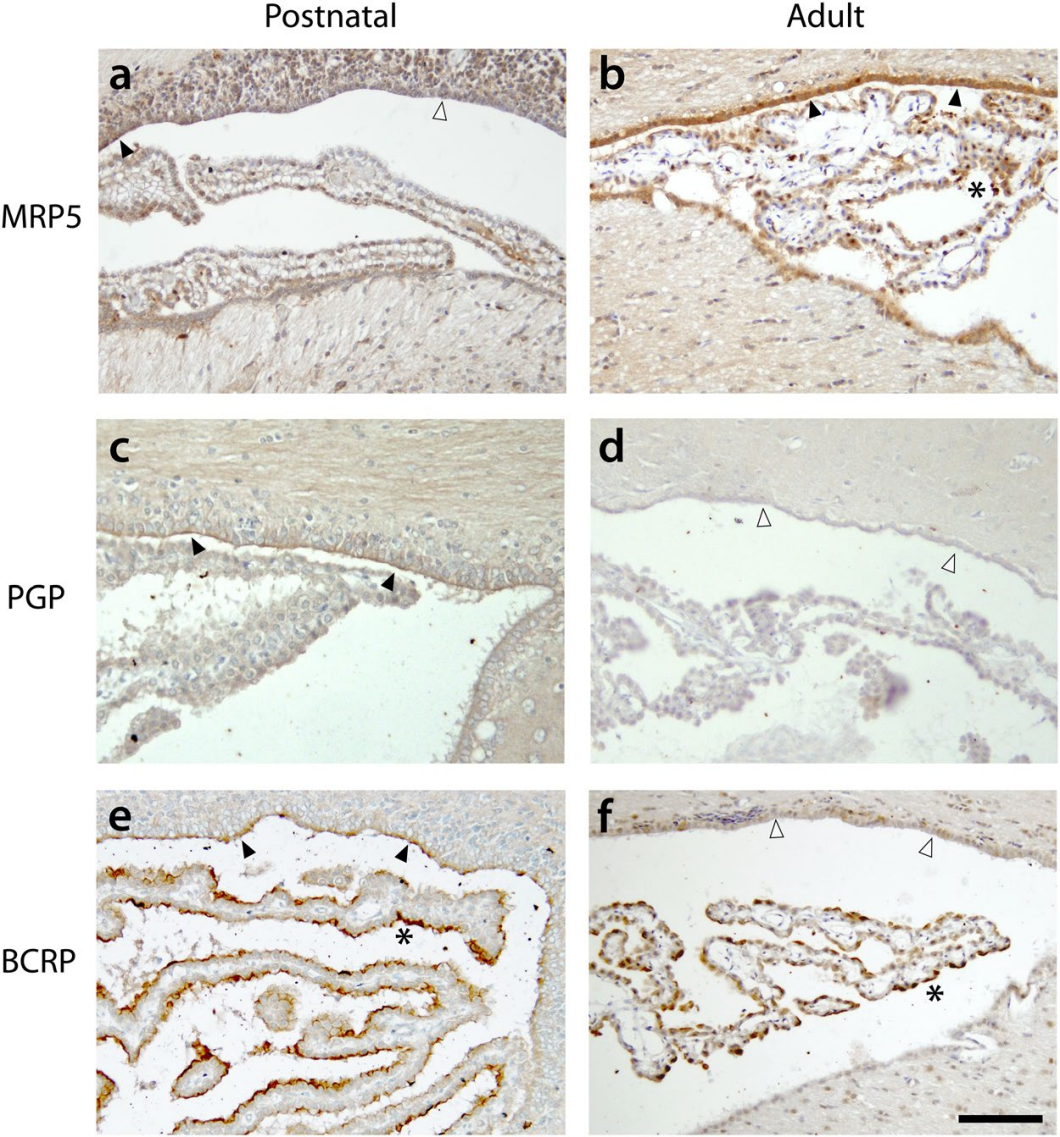
Immunohistochemical staining for ABC transporters at the CSF/brain ventricular interface in the rat. This layer changes from a neuroepithelium in the developing brain to ependyma in the adult. Coronal sections through lateral ventricles of postnatal P4 (**a**), P10-14 (**c**,**e**) and adult cortex (**b**,**d**,**f**). Sections are immunostained with antibodies to: MRP5 (*abcc5*) for (**a**,**b**), PGP (*abcb1a/b*) for (**c**,**d**) or BCRP (*abcg2*) for (**e**,**f**). Dorsal side is up. Note distinct immunostaining, indicated by filled arrowheads, of MRP5 in the adult brain (**b**) and PGP/BCRP in the postnatal brain (**c**,**e**). This is in contrast to the absence of immunostaining, indicated by unfilled arrowheads, of PGP and BCRP in the adult (**d**,**f**). MRP5 staining in the postnatal brain (**a**) is lightly present in some regions, filled arrowheads, and absent in others, unfilled arrowhead. Also note positive immunostaining in choroid plexuses in (**b**,**e**,**f**) (indicated by *). For age-related differences in the immunostaining for other ABC-transporters see Table 1. Scale bar 100 μm (bottom right) applies to all images.

Five-μm thick coronal sections through the cortex from P4, P14 and adult rat brains were immunostained with a battery of antibodies to the ABC transporters investigated for RT-qPCR (see Methods). A summary of the results is compiled in Table 1 and illustrated in Fig. 6 for three transporters: BCRP (*abcg2*), MRP5 (*abcc5*) and PGP (*abcb1a/b*). The immunostaining pattern for different ABC transporters seemed to fall into three general categories: (i) transporters that showed stronger immunostaining at the earlier ages, with minimal or no staining in the adult (PGP; *abcb1a/b* and BCRP; *abcg2*), (ii) transporters whose immunostaining did not appear to change between postnatal ages (MRP3; *abcc3*), and (iii) transporters that showed much stronger staining in the adult compared to younger ages (MRP2, 4 and 5; *abcc2/4/5*). No MRP1 (*abcc1*) staining was observed in the ependymal layer at any age, despite strong staining in the nearby choroid plexus (not illustrated; see Ek *et al*.^9^). It was also noticeable that for all transporters staining was more prevalent along the dorsal ventricular wall compared to ventral. These results indicate that at least some ABC transporters are present at the CSF-brain interface and their differential cellular distribution may relate to their role in brain development (see Discussion).

**Table 1:** A summary of results from the immunohistochemical analysis of ABC transporter distribution at the ventricular neuroepithelial/ependymal interface.

Age	PGP	BCRP	MRP1	MRP2	MRP3	MRP4	MRP5
P4	+	++	−	−	+/−	+	+/−
P14	+	++	−	+	+	+	++
Adult	−	−	−	++	+	++	+++

Animal ages are indicated on the left (P4, P14 and adult). Sections from at least two brains were used in each age group and 4–6 individual sections analysed. Intensity of the staining was scored by two independent observers. − indicates no observable staining, +/− indicates minor staining on some sections and no staining on others, + indicates light but consistent staining, ++ indicates distinct and consistent staining across the ventricular surface, and +++ indicates uniformly strong staining along the whole ventricular layer.

## Discussion

The aim of the present study was to investigate the regulation of ABC transporters and GST enzymes in brain cortices (representing the blood-brain barrier as well as the overall brain protective mechanisms) and choroid plexuses (site of blood-CSF barrier) early in postnatal rat brain development in response to a drug challenge. In addition, we aimed to describe the cellular distribution of these transporters at a CSF-brain interface as this route of entry into the brain has been proposed to be of particular importance in early development^5,23,24^. The results provide information regarding the expression and presence of ABC transporters in three main brain barriers, thus contributing to the understanding of what properties are important to consider for safer drug prescription and future development of therapeutics.

Adult animals used in the present study covered an age-range from 6–10 weeks. There were no statistically significant differences in age or weight between adult groups in all experiments. Previous transcriptomic studies have also shown that over the period of 6–21 weeks of age the only difference in ABC transporters expression is a very minor increase of MRP1 (p = 0.04; FC = 1.05; supplementary dataset)^29^. Some previous studies in rodents have indicated that ABC-transporter expression at blood-brain and blood-CSF interfaces may be different between sexes^30^. However other transcriptomic studies have established that for both P14 and 6 weeks adult rats there are no statistically significant differences in ABC transporter expression in the brain between male and female animals^29^. There appear to be no published studies reporting on the influence of sex on ABC transporter regulation at brain interfaces following xenobiotic challenge. The present study was not designed to look at differences between sexes. Nevertheless, future experiments addressing this would be of interest as well as expanding the study to include the developmental regulation of ABC transporters at the protein and functional levels.

In the present study cortical tissue samples (brain cortices) were used as a measure of the blood-brain barrier expression of ABC transporters and related enzyme. This choice over other techniques, such as endothelial cell separation, was made for a number of reasons. Principally any transporters on cells within the brain will contribute to the prevention of molecular transfer into those cells where they can cause harm, and therefore do not necessarily warrant exclusion. Additionally, questions also remain over how endothelial cell separation techniques may select for some blood vessel types over others^31^; extracted cells may have different properties from different regions of the central nervous system^32,33^ and show altered cell properties in culture, particularly in the case of immortalized cell types^34^. Previous studies already established the cellular distribution profiles of several ABC transporters both in the brain and choroid plexus in the fetal and postnatal rat brain confirming that efflux transporters are largely confined to brain barrier interfaces^9^. Nevertheless it is possible that in the present study small, and/or highly localized changes in expression levels at the blood-brain barrier were not detected because of dilution by brain tissue.

Diallyl sulfide (DAS) was used as the primary inducer based on previous studies identifying it as the most potent inducer, out of a range of compounds tested, of ABC-transporter expression at the blood-brain barrier *in vivo*^12^. The liver was used as a positive internal control. In all age groups chronic DAS (200 mg/kg) exposure resulted in significant up-regulation of hepatic *abcc3* (MRP3) and *abcb1a* (PGP) expression (Fig. 2) as well as GST activity (Fig. 5). As the level of up-regulation in the liver was similar between all ages investigated it is likely that the DAS compound was in the circulation in similar effective quantitates. The observed up-regulation of GST and *abcc3* (MRP3) in the adult liver is consistent with previous findings^25,26,35,36^. The present study expands on these previous adult findings to the early postnatal period (P4 and P14), suggesting similar levels of up-regulation to those observed in the adult. Up-regulation of *abcb1a* (PGP) in the liver at all ages also provides additional information not previously reported. This result contradicts those previously described in the mouse, which suggested no *abcb1a* (PGP) up-regulation in the adult mouse liver^12^. This could be due to species differences or some technical discrepancy (see also below).

Previous studies have shown that GST up-regulation in response to DAS occurs not only in the liver but also in other tissues such as the lung^37^. In the present study no significant up-regulation was observed in the brain or choroid plexus (lateral ventricular or 4^th^ ventricular). The tissue specific differences in up-regulation we observed between liver and brain add to existing evidence of the low induction capacity of metabolizing enzymes in the brain^38^. This may be due to a regulation pathway controlling GST induction by DAS that is more prevalent in tissues other than the brain and choroid plexus. However, various transcription factors including CAR (constitutive androstane receptor) and NRF2 (nuclear factor (erythroid-derived 2)-like 2) linked to the inductive properties of DAS^39^ are well expressed in the choroid plexus^40^. As monochlorobimane is a common substrate for different GST isoenzymes, it is possible that the increase in activity could be due to DAS increasing certain GST isoenzymes in the liver that are not present in the cortex or choroid plexus. The isoenzymatic profile does differ between the liver, cerebral cortex and choroid plexus, with the liver rather immature at early postnatal developmental stages compared to the choroid plexus^17^. Although previous studies suggest that cortical *abcb1a/b* (PGP) up-regulators (dexamethasone and PCN, whose inductive activity is considered to by mediated by the PXR, pregnane X receptor, pathway) also elicit GST up-regulation^17^, this was not the case with DAS in the present study. These results highlight the drug specific differences of up-regulation capacity as well as the multiple responses one drug can elicit on multiple organs.

While DAS did not detectably alter GST conjugating enzyme activity at brain barrier interfaces, it was able to alter the expression of ABC-transporters. This regulation appears to be age-dependent. In the adult up-regulation of cortical ABC-efflux transporters *abcb1a/b* (PGP), *abcg2* (BCRP), *abcc4* (MRP4) and *abcc5* (MRP5) was observed, while earlier in development (P4, P14) this was not the case. DAS had no influence on the major choroid plexus transporter *abcc1* (MRP1), although it did have varying influence on *abcb1a* (PGP) and *abcc2* (MRP2) levels including up-regulation at the P14 age group. It is noteworthy that in adults *abcb1a/b* (PGP) was up-regulated in cortical samples (“blood-brain barrier”) but not in the lateral ventricular choroid plexus. The contrast between cortical up-regulation in adulthood and choroid plexus up-regulation earlier in development (P14) fits with some current suggestions in the literature. It has been proposed that earlier in development the choroid plexus may be the major route of molecular entry into the brain due to factors such as the choroid plexus being developed and functional^23,24^ long before complete vascularization of the brain, which in the rat is approximately P15-P21^41^. The pharmacological significance, however, of *abcb1a* (PGP) and *abcc2* (MRP2) up-regulation in the P14 choroid plexus in this study remains elusive, due to very low choroidal expression of those transporters. Further investigation into the up-regulation capacity of the choroid plexus early in development (directing up-regulation to major transporters *abcc1*; MRP1 or *abcc4*; MRP4) may be valuable in discerning if the above comments are confirmed.

The results obtained from brain cortical and choroid plexus samples provide an interesting comparison with the liver in terms of tissue specific up-regulation. The observed increase in GST activity and *abcb1a* (PGP)/*abcc3* (MRP3) expression in the liver, but not at the brain interfaces of most ages is consistent with other studies that have reported tissue specific responses to xenobiotics^12,42^. Additionally the RT-qPCR results from the adult indicate that up-regulation at one of the blood-brain interfaces does not mean up-regulation will occur at all of them. This highlights the importance for regulation studies to investigate changes at multiple blood-brain interface sites, as it is likely that not all respond in the same way.

In the early postnatal period (P4) chronic DAS exposure resulted in down-regulation of ABC transporters in the cortex and choroid plexus. While it was hypothesized that the developing brain interfaces may have less capacity to up-regulate efflux transporters to the same extent as adults, down-regulation was an unexpected finding. This is the first example of the developing brain barriers actually being reduced in effectiveness by repeat drug exposure, potentially leading to a weaker level of defences than we would see following acute administration. This result, combined with an increase in adult defences, may have major implications for our understanding of safe medication doses during pregnancy. Over time a mother taking a neurologically targeted medication may find the same dose becoming less effective for her (as efflux transporters up-regulate) while there is increased drug access to her baby’s brain (as their defences down-regulate). For different compounds the opposite may also occur; in sertraline treated pregnant mice *abcb1* (PGP) expression increased in the placenta but decreased in both the fetal and maternal blood-brain barriers^43^. Future studies investigating a range of medications would assist in determining whether the result observed in this study is due to a developmentally specific toxic effect of DAS or to an age-specificity in regulation capacity for a range of compounds.

The cortical samples collected for RT-qPCR analysis in this study included the CSF-brain interface at the level of the ventricular neuroepithelium in postnatal animals and ependyma in adults. In the early developing brain, cells that line the ventricular system are connected by “strap” junctions first described in 1987 in sheep fetuses^22^. The function of these junctions appears to be to prevent free diffusion of lipid insoluble molecules between CSF and brain parenchyma. As development progresses these junctional complexes are replaced by gap junctions resulting in the adult ependymal layer, which does not pose a diffusional restrain^44^. Functional studies in the developing rat have shown that these junctional complexes restrict the CSF-to-brain transfer of compounds up to 3 kDa from embryonic stages until P10^44^. At P10 entry of 10 kDa size proteins begins to occur, with up to 70 kDa compounds entering from P20 onwards^44^. This means that in the present study it could be expected that P4 and P14 animals would still have a restrictive CSF-to-brain barrier interface present. While “strap” junctions form a development-specific barrier to intercellular transfer, whether or not ABC efflux transporters are also present to restrict the exchange of lipid soluble compounds at this interface has not yet been conclusively described. Any changes in ABC transporter expression at this interface during development would be critical to molecular transfer into the brain. Daood and colleagues^28^ identified MRP1 (*abcc1*) at the ventricular barrier suggesting constant levels over human development, whereas Tomioka and colleagues^27^ reported no BCRP (*abcg2*) immunostaining in the adult mouse ependyma. Determining whether or not each transporter is present at the CSF-brain interface would be important both in interpreting RT-qPCR results (as the layer contributed to the gene pool of cortical samples) and in understanding molecular transfer between the CSF and brain over development.

RT-qPCR analysis in the present study revealed that *abcb1a/b* (PGP), *abcg2* (BCRP) and *abcc4/5* (MRP4/5) were up-regulated following DAS exposure in the adult rat cortex, but not earlier in development. Immunohistochemical analysis indicated that PGP (*abcb1a/b*) and BCRP (*abcg2*) were not present at the adult CSF-brain interface, suggesting that the RT-qPCR cortical up-regulation of these transporters was unlikely to be due to changes at this interface. In contrast, MRP4/5 (*abcc4/5*) were present at the adult CSF-brain ependymal layer, suggesting that mRNA obtained from cells forming this interface may have contributed to the overall cortical RT-qPCR results. These transporters were, however, also present at the CSF-brain interface earlier in development (P4, P14) when no RT-qPCR up-regulation was observed in cortical samples.

The present study has demonstrated that there are many efflux transporters present at the CSF-brain interface that could act to prevent or limit drug exchange between the CSF and the brain. As outlined in and Fig. 6 and Table 1 the intensity of the immunostaining for individual transporters appears to change during development. Immunostaining for PGP (*abcb1a/b*) and BCRP (*abcg2*) was only detected at P4 and 14 and not in the adult, while strong MRP2/4/5 (*abcc2; abcc4; abcc5*) staining was observed mostly in the adult ependyma. The lack of BCRP (*abcg2*) staining in the adult corresponds with reports by Tomioka and colleagues^27^ in the adult mouse, but our results suggest that earlier in development the transporter is present. An interesting aspect of the present results is the strong MRP2-5 (*abcc2-5*) staining at the ependymal layer in the adults. Exchange between the CSF and the brain in the adult is unrestricted as gap junctions connect ependymal cells^22,44^ allowing intercellular transfer. It is therefore possible that transporters such as MRP2/4/5 (*abcc2; abcc4; abcc5*) are responsible for removing compounds from the brain into the CSF rather than preventing transfer the other way. These transporters may allow the efflux of glutathione-conjugates formed in the CSF-brain interface by multiple isoenzymes that have been shown to exist at this barrier during development and adulthood^17^. The disappearance of “strap” junctions and expansion of gap junctions associated with the transformation of neuroepithelium to ependyma together with the increase in MRP transporters would, theoretically, work together to provide enhanced clearance from the brain to CSF in adults compared to early in development. The contribution of a drainage system, such as the recently described glymphatics^45,46^, to drawing compounds towards the brain-CSF exchange surface is yet to be investigated. The presence of PGP (*abcb1a/b*) and BCRP (*abcg2*) in the neuroepithelium at P4 and P14, however, could indicate that these transporters are preventing the unrestricted access of CSF-borne compounds into the brain early in development.

## Conclusion

This study has expanded our knowledge of the defence capacity of the blood-brain interfaces over different developmental stages, contributing to a greater understanding of the mechanisms influencing the transfer of xenobiotic compounds into an immature brain. The ability of different blood-to-brain exchange interfaces (blood-brain barrier contained within the cortices and the blood-CSF barrier at the choroid plexuses) to dynamically regulate ABC-transporters’ expression has been investigated, revealing both tissue-specific and age-specific differences in regulation. Results from this study are consistent with the proposition that choroid plexuses play a prominent role in the regulation of blood to brain exchange early in development when it has a larger impact on brain transfer, compared to the blood-brain barrier which mostly regulates defences once the brain is fully vascularized. The profiles of cellular distribution of ABC-transporters at the ventricular interface are described, with PGP (*abcb1a*) and BCRP (*abcg2*) present early in development and strong MRP2-5 (*abcc2-5*) immunostaining present in adulthood, supporting the importance of this interface in the immature brain. Expansion of these results to studies of a wider range of drugs and drug classes would provide critical information to pharmaceutical companies and to medical practitioners who prescribe medications to pregnant women and neonates.

## Materials and Methods

### Gene Expression Studies (RT-qPCR)

#### Animals

All procedures involving animals were approved by the University of Melbourne Animal Ethics Committee and conducted in compliance with Australian National Health and Medical Research Guidelines. Sprague Dawley rats were supplied by the University of Melbourne Biological Research Facility and housed in groups of 2–4 (adult) or full litters per cage, on a 12 h light/dark cycle with *ad libitum* access to food and water. Age groups investigated (listed as treatment completion) were P4, P14 and adults (6–10 weeks). Postnatal litters were both males and females, while adults were females only. Rats from each age were randomly assigned to either DAS treated or control groups (n per group depicted in figure legends).

#### Treatment regime

Treated animals (referred as the chronically treated group) received 200 mg/kg of undiluted diallyl sulfide, DAS (SIGMA, A35801). Injections were given intraperitoneally (i.p.) once daily for 4 days with tissue taken on the 5^th^ day, as described by Cui *et al*.^12^. Age-matched control animals received no injections.

#### Tissue Collection

**Figure 7:**
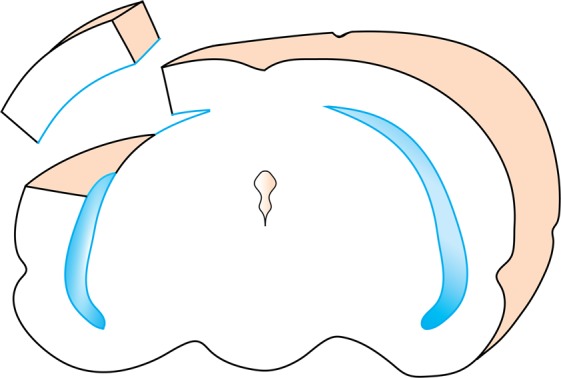
Schematic diagram illustrating the position of the cortical segment collected for RT-qPCR, GST assay measurements and immunohistochemical analysis. The segment was dissected out from above the ventricle (outlined in blue) and included the ventricular layer (neuroepithelial or ependymal depending on developmental age). All pia mater and choroid plexuses were removed prior to brain sampling.

At the end of the treatment regime animals were killed with an overdose of inhaled isoflurane followed by cardiac transection. Tissue samples were extracted under RNAse free conditions into cryovials and immediately frozen in liquid nitrogen and stored at −80 °C. Choroid plexuses (lateral ventricular) were removed from brains prior to cortex tissue sampling. Cortex samples were collected as a standard block of brain tissue dissected out above the ventricle of mainly frontal and parietal lobe tissue (see Fig. 7 for a diagram illustrating the position of cortex sampling).

#### RNA extraction and cDNA conversion

RNA was extracted using commercially available RNeasy Plus Mini Kits (Qiagen) and QIAshredder (Qiagen) for liver and cortex or using the RNeasy Plus Micro Kits (Qiagen) for choroid plexus, as per manufacturers specifications. RNA quantity and purity was determined using a NanoDrop ND-1000 UV-VIS spectrophotometer (Thermo Scientific). cDNA conversion was conducted using the Applied Biosystems High Capacity RNA-cDNA Kits with each sample containing 20 μl (10 μl 2x reverse transcriptase buffer mix, 1 μl 20x RT enzyme mix and 9 μl combination of RNA and nuclease free water to standardize sample concentrations). Samples were incubated in a thermocycler (60 mins at 35 °C, 5 mins at 95 °C and 4 °C until removed) and cDNA stored at −20 °C until use.

#### Real-time quantitative polymerase chain reaction (RT-qPCR)

The primers used for RT-qPCR gene expression measurements are shown in Table 2 and were pre-validated for single peak melt curves and efficiencies of 90–100%. Measurements were conducted on 10 μl wells using Rt^2^ SYBR Green ROX qPCR Mastermix (Qiagen) on a ABI Prism 7900HT Real Time sequence analyzer (Applied Biosystems) as follows: 50 °C (2 min), 95 °C (10 min), 40 cycles of 95 °C (30 sec), 60 °C (1 min). No-template controls were run simultaneously and gave no signal (or occasional negligible signal >38Ct). Threshold cycle time (Ct) values were obtained as an average from triplicates for each gene. Relative gene expression was calculated as a comparison between the gene of interest and an average of two housekeepers (β-actin and cyclophilin b; peptidylprolyl isomerase B; ppib) and expressed as 2^−ΔCt^ (2^−(GeneCt-HousekeeperCt)^). Each dot in the figures represents an individual animal. Each age was run on a single RT-qPCR plate (with all transporters) and results combined for graphical representation.

**Table 2:** List of the RT-qPCR primer sequences and the associated NCBI sequences.

Target	Forward	Reverse	NCBI
β*-actin*	*CCCTAGACTTCGAGCAAGAG*	*GGATTCCATACCCAGGAAGG*	NM_031144.3
*ppib*	*AGTGACCTTTGGACTCTTTGG*	*TCCTTGATGACACGATGGAAC*	NM_022536.2
*abcb1a*	*CAACCAGCATTCTCCATAATA*	*CCCAAGGATCAGGAACAATA*	NM_133401.1
*abcb1b*	*CCATGTGGGCAAAGGTACTGA*	*CTAAGACTTCTTCGGCAACT*	NM_012623.2
*abcg2*	*CAGCAGGTTACCACTGTGAG*	*TTCCCCTCTGTTTAACATTACA*	NM_181381.2
*abcc1*	*CCTTGGGTCTGGTTTACTT*	*ACAGGGGAACGACTGACAG*	NM_022281.2
*abcc2*	*CAGGGCTGTGCTTCGAAAATCCAAAA*	*GTGTGCAGCCTGTGAGCGATGGTGAT*	NM_012833.2
*abcc3*	*CTCGCCCATCTTCTCCCACTTCTCGG*	*CCGGTTGGAGGCGATGTAAGGATAAG*	NM_080581.1
*abcc4*	*GAACGCTACGAGAAAGTCATC*	*GCCCGTGCCAAGTTCAC*	NM_133411.1
*abcc5*	*AACAGGAAGGATTCTCAACAGG*	*TGAATGCTGGACGTGATATGG*	NM_053924.1

### GST Activity Assay

#### Animals

All procedures involving animals were approved by the French Ethical Committee (decret 87–848) and conducted in compliance with the European community directive (86-609-EEC). Sprague Dawley rats were supplied by Janvier Labs and housed in groups of 2–4 (adult) or single female with litter per cage, on a 12 h light/dark cycle with *ad libitum* access to food and water. Age groups (at treatment completion) included P4 pups (males and females) and 6–10 week-old adults (males).

#### Treatment regime

Treated animals received 200 mg/kg of undiluted diallyl sulfide, DAS (SIGMA) per injection. Injections were given i.p. once daily for 3 days with tissue taken on the 4^th^ day as per previous protocols regarding NRF2 and CAR pathway induction of GST^47,48^. Age-matched control animals received no injections.

#### Tissue Collection

Animals were sacrificed by decapitation following isoflurane anesthesia. Tissue (liver, lateral and 4^th^ ventricular choroid plexus, cerebral cortex) was extracted on ice and stored at −80 °C. Choroid plexuses and meningeal tissue were removed from brains prior to cortex tissue sampling to ensure that no choroid plexus tissue was present in cortex measurements (Fig. 7).

#### GST Assay conditions

GST specific activity was measured using the GST isoform multispecific substrate monochlorobimane (MCB; Sigma) as previously described^17^. Briefly, tissues were kept on ice and glass-glass homogenized in a buffer (0.25 M sucrose, 50 nM K-phosphate, 1 mM EDTA, 0.1 mM DTT, pH 7.4). Homogenized samples were added in varying amounts to reaction buffer (10 mM K-phosphate, 0.02% BSA, pH 6.5) with 50 μM MCB and 1 mM glutathione (GSH; Sigma). The glutathione conjugate formed, MCB-SG, was measured spectrofluorimetrically by using kinetic analysis with excitation/emission wavelengths of 355/460 nm (Tecan Infinite M200 Pro). The linearity of fluorescence rates as a function of protein content in the assay was verified for each sample. Assays in the absence of homogenates were also run as controls to account for non-enzymatic conjugation.

The specific activity was calculated using a GSMCB standard prepared exactly as previously described^17^, and normalized for total protein content. Total protein content was measured by spectrophotometry using the Peterson^49^ method in a CARY 100 scan spectrophotometer set at 750 nm. A calibration curve was created using bovine serum albumin as a standard.

#### Immunohistochemistry

Bouin’s fixed, paraffin embedded five micron coronal sections of control rat brains were selected from serially sectioned tissue obtained from another study^50,51^. Sections were selected to correspond to the ages and brain region used for RT-qPCR (see above). At least two brains from each age group (P0-4 referred to as P4; P10-14 referred to as P14; adult: 180–200 g females) were included in the study.

Selected sections were de-paraffinized in xylene and rehydrated in graded ethanol following standard procedures. For some antibodies the antigen retrieval step was applied. This involved boiling sections for 10 min in either citrate buffer (pH6) or TEG (1.211 g Calbiochem Tris base, 0.190 g SIGMA EGTA, 1 L distilled H_2_O, pH9). All sections were incubated with 0.5% hydrogen peroxide in TBS (Tris-buffered saline: 5 mM Tris-HCL, 146 mM NaCl, pH7.6) to quench endogenous peroxidase and in 10% normal goat serum for 30 min to block non-specific binding sites. Primary antibodies (listed with relevant details in Table 3) were incubated on sections overnight at 4 °C. Antibodies were diluted in 10% normal goat serum in TBS. Following extensive washings in TBS, REAL EnVision Detection System (Peroxidase/DAB + rabbit/mouse, code K5007, DakoCytomation, Denmark) was applied. The positive reaction product was visualised by incubating sections, washed again with TBS, for 10 min with the DAB + detection kit (DakoCytomation, Denmark). Positive staining appeared as dark brown deposit. Sections were washed (TBS), counter stained with Mayer’s hematoxylin (Sigma), dehydrated through graded ethanol and xylene and cover-slipped with Pertex mounting medium (HistoLab). Control sections were either incubated with unrelated rabbit/mouse immunoglobulin or for negative controls the primary antibody was omitted and these sections were always blank. Sections were photographed under bright field using an Olympus BX50 microscope (fitted with a DP60 digital camera) with minimal color manipulation used only to increase the contrast.

**Table 3:** List of the primary antibodies used for immunohistochemical staining.

Primary Antibody	Host IgG	Dilution	Retrieval	Supplier	Code Number
PGP (*abcb1*)	Mouse IgG1	1:15–1:30	TEG	Abcam	ab3366
BCRP (*abcg2*)	Mouse IgG2a	1:15–1:20	M6	Abcam	ab3380
MRP1 (*abcc1*)	Mouse IgG2a	1:30–1:50	M6	Abcam	ab24102
MRP2 (*abcc2*)	Rabbit IgG	1:200	—	Bioss	bs-1092R
MRP3 (*abcc3*)	Rabbit IgG	1:100	—	Bioss	bs-10163R
MRP4 (*abcc4*)	Rabbit IgG	1:100	—	Abcam	ab180712
MRP5 (*abcc5*)	Rabbit IgG	1:100	—	Abcam	ab180724

### Statistics

Statistical significance was determined by 2-tailed students t-test. All data were tested for equal variance using an f-test and for normality using the Shapiro Wilk test (with a Mann-Whitney U correction if required).

## Supplementary information

Supplementary information

## Data Availability

Data from this study are included in this published article (and its Supplementary Information files). Any further information is available from the corresponding author on request.
