# Supplementary material for: Developmental differences in the expression of ABC transporters at rat brain barrier interfaces following chronic exposure to diallyl sulfide
Source: Sci Rep. 2019 Apr 12;9:5998. doi: 10.1038/s41598-019-42402-8 (PMC6461637; doi:10.1038/s41598-019-42402-8)
Supplement: Supplementary file 1 — Supplementary information [file 41598_2019_42402_MOESM1_ESM.pdf]

# Supplementary Material

# Developmental differences in the expression of ABC transporters at rat brain barrier interfaces following chronic exposure to diallyl sulfide

Liam M Koehn[1], Katarzyna M Dziegielewska[1], Kjeld Møllgård[2], Elodie Saudrais[3], Nathalie Strazielle[3,4], Jean-Francois Ghersi-Egea[3], Norman R Saunders[1], Mark D Habgood[1]

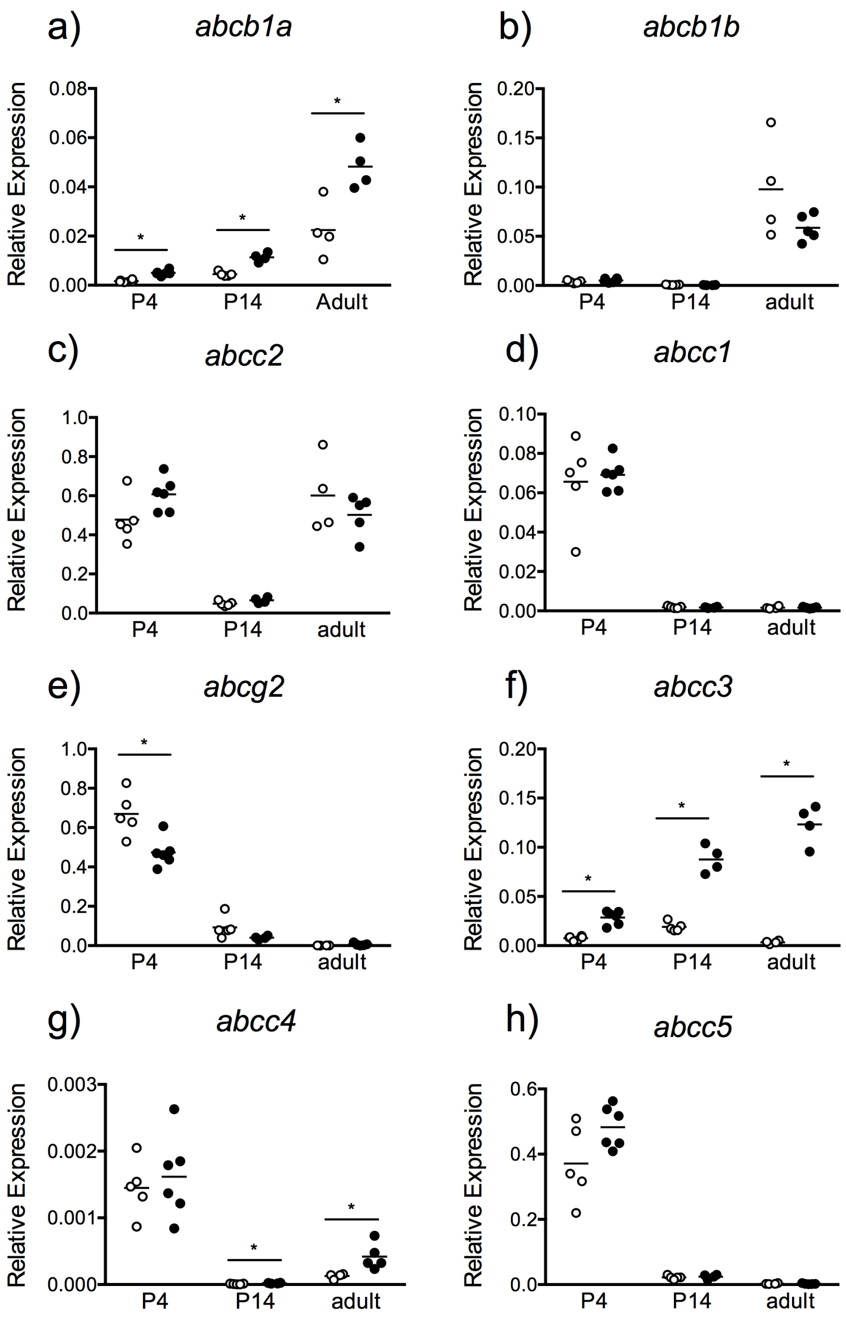


**Figure S1.** Hepatic expression of (**a**) *abcb1a* (PGP), (**b**) *abcb1b* (PGP), (**c**) *abcg2* (BCRP), (**d**) *abcc1* (MRP1), (**e**) *abcc2* (MRP2), (**f**) *abcc3* (MRP3), (**g**) *abcc4* (MRP4), (**h**) *abcc5* (MRP5) for both untreated control animals (open symbols) and chronically DAS treated (200mg/kg; filled symbols) animals. Relative expression was calculated as $2^{-\Delta Ct}$ with respect to an average of two housekeeping genes: *β-actin* and *ppib*. Ages investigated were P4, P14 and adult, referring to the age when tissue was collected following the completion of the treatment protocol. * indicates a significant difference ($p<0.05$) between treatment groups for each age and transporter. Note that different ages were run on different plates and combined for graphical representation. Also note the different scales on the y-axes.

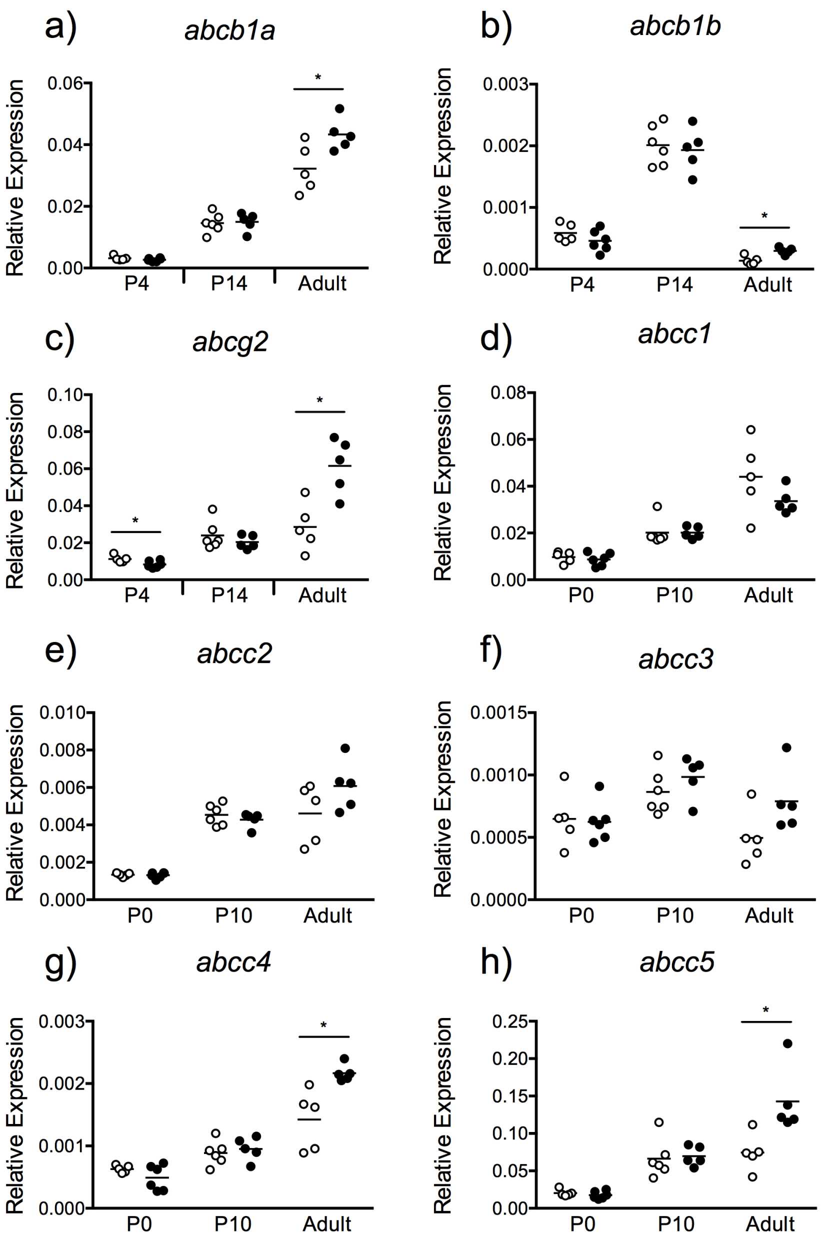


**Figure S2.** Brain cortical expression of (**a**) *abcb1a* (PGP), (**b**) *abcb1b* (PGP), (**c**) *abcg2* (BCRP), (**d**) *abcc1* (MRP1), (**e**) *abcc2* (MRP2), (**f**) *abcc3* (MRP3), (**g**) *abcc4* (MRP4), (**h**) *abcc5* (MRP5) for both untreated control animals (open symbols) and chronically DAS treated (200mg/kg; filled symbols) animals. Relative expression was calculated as $2^{-\Delta Ct}$ with respect to an average of two housekeeping genes: *β-actin* and *ppib*. Ages investigated were P4, P14 and adult, referring to the age when tissue was collected following the completion of the treatment protocol. * indicates a significant difference ($p<0.05$) between treatment groups for each age and transporter. Note that different were ages run on different plates and combined for graphical representation. Also note the different scales on the y-axes.

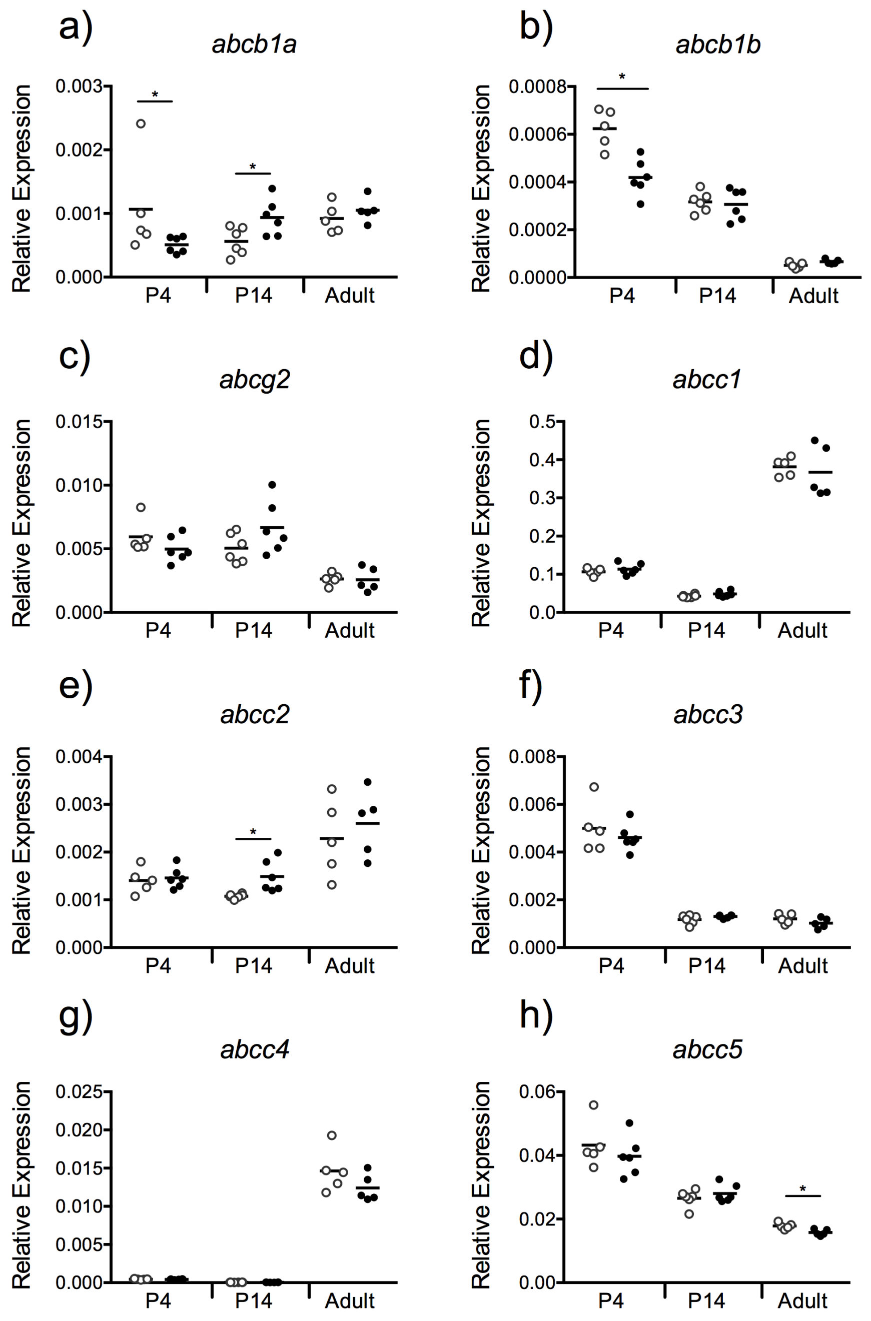


**Figure S3.** Lateral ventricular choroid plexus expression of (**a**) *abcb1a* (PGP), (**b**) *abcb1b* (PGP), (**c**) *abcg2* (BCRP), (**d**) *abcc1* (MRP1), (**e**) *abcc2* (MRP2), (**f**) *abcc3* (MRP3), (**g**) *abcc4* (MRP4), (**h**) *abcc5* (MRP5) for both untreated control animals (open symbols) and chronically DAS treated (200mg/kg; filled symbols) animals. Relative expression was calculated as $2^{-\Delta Ct}$ with respect to an average of two housekeeping genes: *β-actin* and *ppib*. Ages investigated were P4, P14 and adult, referring to the age when tissue was collected following the completion of the treatment protocol. * indicates a significant difference ($p<0.05$) between treatment groups for each age and transporter. Note that different ages were run on different plates and combined for graphical representation. Also note the different scales on the y-axes.
